# Performance of loop-mediated isothermal amplification assay in the diagnosis of pulmonary tuberculosis in a high prevalence TB/HIV rural setting in Uganda

**DOI:** 10.1186/s12879-018-2992-1

**Published:** 2018-02-21

**Authors:** Lydia Nakiyingi, Prossy Nakanwagi, Jessica Briggs, Tifu Agaba, Frank Mubiru, Mark Mugenyi, Willy Ssengooba, Moses L. Joloba, Yukari C. Manabe

**Affiliations:** 1 Infectious Diseases Institute, Makerere University College of Health Sciences, P.O. Box, 7072, Kampala, Uganda; 2 Department of Medicine, School of Medicine, Makerere University College of Heath Sciences, Kampala, Uganda; 3 Baylor College of Medicine Children’s Foundation of Uganda, Kampala, Uganda; 4 Division of Infectious Diseases, Department of Medicine, University of California, San Francisco, USA; 5 National Tuberculosis and Leprosy Program, Kiboga District Hospital, Kiboga, Uganda; 6 Department of Microbiology, School of Biomedical Sciences, Makerere University College of Heath Sciences, Kampala, Uganda; 7 Division of Infectious Diseases, Department of Medicine, Johns Hopkins University , Baltimore, USA

**Keywords:** Tuberculosis, Microscopy, LAMP, Diagnostic, Rural, Uganda

## Abstract

**Background:**

Smear microscopy lacks sensitivity especially in HIV co-infection, resulting in undiagnosed tuberculosis (TB) and high mortality. The loop-mediated isothermal amplification (TB-LAMP) assay can be staged with minimal infrastructure, is rapid, low cost and detection can be with the naked eye. We assessed feasibility and performance of Eiken TB-LAMP test at point-of-need in TB diagnosis in a high prevalence TB/HIV rural setting in Uganda.

**Methods:**

From October 2013-February 2014, TB-LAMP testing was performed on sputum specimens from outpatient presumptive TB adults at a district hospital and two low-level health centers in Kiboga District where smear microscopy is the available routine diagnostic option. TB-LAMP was performed by a technician after a week of training in the district hospital. The technician had no prior experience in the technology. Samples from the low-level health centers were transported to the district hospital for TB-LAMP.

**Results:**

Of the 233 presumptive TB (126 at hospital); 113 (48.5%) were HIV-infected; 129 (55%) male; median age 40 (IQR 30-53). Compared to MTB culture, overall sensitivity and specificity of TB-LAMP were 55.4% (95 CI 44.1-66.3) and 98.0% (95 CI 94.3-99.6) respectively. Among HIV-infected participants, TB-LAMP sensitivity and specificity were 52.3% (95 CI 36.7-67.5%) and 97.1% (95 CI 89.9-99.6) respectively; and 24.4% (95% CI 12.9-39.5) and 98.6% (95% CI 95.1-99.8) respectively among smear-negatives. TB-LAMP sensitivity and specificity were 62.2% (95% CI 44.8-77.5) and 97.8% (95% CI 92.1-99.7) in the hospital setting where central testing occurred compared to 50.0% (95% CI 34.9-65.1) and 98.4% (95% CI 91.2-100) respectively in low-level health centers where specimens were transported centrally.

**Conclusions:**

In this high prevalence TB/HIV rural setting, TB-LAMP performs better than conventional smear microscopy in diagnosis of MTB among presumptive TB patients although the sensitivity is lower than that reported by the World Health Organization. TB-LAMP can easily be performed following a short training period and in absence of sophisticated infrastructure and expertise.

## Background

Tuberculosis (TB) is underdiagnosed in most resource-limited settings (RLS) and threatens global TB control; current accurate diagnostics are expensive, limiting availability [1]. Conventional TB diagnostics lack sensitivity resulting in high mortality. Smear microscopy remains the main method for diagnosis in most high prevalence TB/HIV RLS, despite its low sensitivity [2, 3]. Smear microscopy detects less than half of TB cases with lower sensitivity in HIV co-infection [2–5]. The World Health Organization (WHO) recommended rapid tests [1] with better sensitivity such as Xpert MTB/RIF test can only be implemented at sites with electricity or solar panel infrastructure and the operational requirements makes it impracticable in most rural settings [2, 3, 6–8]. The gold standard for diagnosis, TB culture, on the other hand, has a very long turn-around time up to 6 weeks for liquid TB cultures and 8 weeks for solid TB cultures and requires high biosafety standards, limiting the use of TB culture in routine patient management [2, 9]. New tests which may specifically supplement or replace the existing insensitive tests are warranted.

A molecular amplification test method based on loop-mediated isothermal amplification assay (henceforth called TB-LAMP test) was developed with characteristics that allow its use with less sophisticated infrastructure [10–13]. Details of TB- LAMP test with the region of amplification and primers have been described in earlier reports [13]. Briefly, the fundamental reaction requires four types of primer which are complementary to six regions of the target gene: the F3c,F2c and F1c regions at the 3’side and the B1,B2 and B3 regions at the 5’side.The FIP primer consists of the F2 region that is complementary to the F2c region on the target gene at the 3’end, linked to a copy of the F1c target gene sequence at the 5’end. The BIP primer consists of the B2 region that is complementary to the B2c region on the target gene at the 3′ end, linked to a copy of the B1c target gene sequence at the 5’end. The FIP and BIP primers are responsible for the formation of loop structures. F3 and B3 are forward and backward displacement primers complementary to the F3c and B3c regions on the target gene, respectively [13]. TB-LAMP has advantages of rapidity (turn-around time 2 h), ease of application, low cost and a result that can be detected with the naked eye [10–15]. TB-LAMP result is also easily interpretable as it provides a yes or no answer for case detection. With these advantages over other molecular tests, TB-LAMP could be a true point-of-need nucleic acid amplification system which can easily be applied at peripheral laboratories [10–15].

Commercial TB-LAMP manual assays that can be performed with minimal infrastructure requirements and relative ease have been developed by Eiken Chemical Company Ltd. (Tokyo, Japan) in collaboration with FIND for detection of Mycobacterium tuberculosis (MTB) complex [10–13, 15–17]. In a recent report from a multicenter feasibility study on a commercial TB-LAMP test conducted in India, Uganda, and Peru [16], overall sensitivity among culture-positives was 97.2% for smear-positive and 62.0% for smear-negative TB, although the sensitivity varied widely by country and operator. The specificity of the TB-LAMP ranged from 94.5% to 98.0% by country [16].

The WHO recently endorsed TB-LAMP for use as a replacement to smear microscopy or as a follow-on test to smear microscopy in smear-negative specimens for diagnosis of pulmonary TB in adults [10]. However, prior to the widespread roll-out of the TB-LAMP assay, feasibility studies should be conducted in rural settings where the test is intended for use, that is, settings where conventional smear microscopy is still the only TB diagnostic option.

We sought to determine the feasibility and performance of a modified Eiken TB-LAMP test performed in a rural peripheral setting in Uganda with high rates of TB and HIV co-infection compared to liquid and solid MTB cultures. We also compared the performance of the Eiken TB-LAMP test to smear microscopy and Xpert MTB/RIF test using MTB culture as the reference standard. We hypothesized that TB-LAMP used at the intended settings of use (microscopy setting) would have sensitivity higher than smear microscopy and be easily implemented at a District Hospital with diagnostic hubbing from two Health Center III’s.

## Methods

### Study design and setting

A cross-sectional diagnostic study was conducted in Kiboga district, Uganda with participants from Kiboga district hospital and two low-level health centers (Bukomero and Ntwetwe health center III’s) between October 2013 and February 2014. Kiboga district is a rural district in Uganda located at approximately 125 km from Kampala with frequent electricity power cutoffs. The district has a high TB/HIV prevalence with relatively low rates of drug-resistant TB. Smear microscopy is the only diagnostic option and laboratory technicians do not have experience and training in the use of molecular tests.

### Population and sample acquisition

Left-over spontaneously expectorated sputum specimens submitted to the laboratory for TB testing from adult presumptive TB patients as per the WHO criteria [1] aged ≥18 years were used. A patient was designated presumptive TB if he or she reported having cough for 2 or more weeks and presence of any one or more of the constitutional symptoms i.e. fever, night sweats, or weight loss. The sputum was ordered by non-study clinicians during routine care. Two spot specimens are routinely collected for TB investigations in these clinics and the left-over sputum specimens from these collections were used in the study. Specimens obtained from patients that had taken anti-TB medication for more than 2 days within 60 days prior to collection were excluded. Left over specimens considered inadequate (less than 2 ml) for study tests were also excluded. Samples selected for the study were de-identified by non-study staff at the time final routine (non-study) results were completed. Routine mycobacterial test results, particularly smear-microscopy (fluorescence microscopy or Ziehl-Neelson), were recorded for study purposes and linked to the results of the study tests that included: Xpert MTB/Rif test, solid and liquid mycobacterial cultures as well as TB-LAMP test.

Study staff filled a presumptive TB form that contained de-identified clinical information including TB symptoms (including but not restricted to fever, cough, night sweats, weight loss), socio-demographic information (including age, sex and address) and medical history (HIV sero-status, previous TB, current medication including TB medications and anti-retroviral therapy).

### Laboratory procedures

TB-LAMP testing was performed at Kiboga Hospital on the left-over sputum specimens. All transportation of sputum samples from low-level Health centers to Kiboga District Hospital and then to Kampala was via a motor vehicle and involved use of cooler boxes. All specimens were tested immediately upon receipt in the laboratories or kept at 4^0^ C before testing for a maximum of 12 h after receipt. Sputum specimens intended for MTB culture were transported from Kiboga to Kampala within 24 h with the intention to have all specimens tested within approximately 24 h of being obtained. Xpert MTB/Rif test was performed at the Infectious Diseases Institute (IDI) laboratory in Kampala due to unavailability of Xpert MTB/Rif testing in Kiboga Hospital at the time of the study. Sputum TB culture was performed at the College of American Pathologist (CAP) accredited Mycobacteriology biosafety level-3 (BSL-3) laboratory under Makerere University, Department of Medical Microbiology located within the Mulago Hospital complex, Kampala.

### TB-LAMP procedure

A modified Eiken TB-LAMP assay with sample preparation and isothermal amplification was performed during the study. The modified Eiken TB-LAMP assay which has a procedure for ultra-rapid DNA extraction (PURE-LAMP) [17, 18] has a simplified specimen processing methodology which simplifies the procedure and has two amplification targets, i.e. IS6110 and gyrB unlike the earlier versions [12, 13] which had only gyrB as the amplification target. These features make the modified Eiken TB-LAMP assay more sensitive and feasible in peripheral centers since raw sputum can be used without need for pre-concentration steps or special biosafety measures [10, 17, 18].

Prior to the study, two laboratory technicians who did not have experience in molecular tests received 1 week of training on how to perform the TB-LAMP test. Laboratory staff competency in correct and accurate performance of the TB-LAMP assay was assured prior to the beginning of the study through proficiency testing. The training on TB-LAMP assay was provided by Eiken Chemical Company. Ultimately, the TB-LAMP procedure was performed by a single technician. TB-LAMP testing for all specimens was performed at Kiboga hospital in a simple room without a biosafety cabinet that was prepared by the laboratory technician who performed the procedure. Samples from the low-level health centers were transported to Kiboga hospital for testing that was done on the same day. Briefly, the TB-LAMP procedure whose detailed description can be found in earlier reports [10, 13, 16–18] involved sputum processing, DNA extraction, isothermal amplification and visual readout with ultraviolet (UV) fluorescence. In each run, a positive control and a negative control were included. The TB-LAMP test results were interpreted according to the following criteria: Positive if any fluorescence present (except if negative control is positive), negative if no fluorescence present and indeterminate if reader is not able to decide on the presence of fluorescence. Interpretation of contaminated sample was assumed if a false-positive negative control was obtained.

Information on operational suitability of TB-LAMP assay for use at the site was obtained from the laboratory study personnel who performed the TB-LAMP test by use of a standardized questionnaire.

### Xpert MTB/Rif and MTB culture testing

Sputum specimens from Kiboga District were received at the Makerere TB laboratory where sample preparation was performed immediately or stored at 4 °C. The sputum specimens were decontaminated using N-acetyl-L-cysteine- sodium hydroxide (NaOH 1.5% final concentration) of which 0.5 ml was used to inoculate Mycobacteria Growth Indicator Tube (MGIT) culture using the MGIT 960 system (Becton and Dickinson, Franklin Lakes, NJ USA) and 0.1 ml to inoculate Lowenstein-Jensen (LJ) culture media. Cultures were incubated at 37 °C for up to 6 weeks for MGIT and 8 weeks for the LJ method. Positive cultures were assessed for presence of acid-fast bacilli (AFB) using ZN staining and light microscopy, and for *M. tuberculosis* (MTB) complex using an anti MPB64 antibody assay (Capilia TB-Neo, TAUNS Laboratories, Numazu, Japan). Drug susceptibility testing to (at least) isoniazid and rifampin was performed on one positive culture from each participant having culture-positive TB. The remaining portion of each decontaminated sputum sample was transported to the IDI for Xpert MTB/Rif testing.

HIV results from the routine HIV clinic were recorded at the time of sample submission and considered for this study. HIV tests in Kiboga district health centers are performed using methods approved and provided by the Uganda Ministry of Health [19].

All study TB laboratory results except for the TB-LAMP that was an investigational test were made available to the attending clinicians at the study sites. TB treatment for TB patients was prescribed by the attending clinicians at the study sites and was according to the guidelines from the Uganda Ministry of Health TB and Leprosy program [20].

### Data management and statistics

Data was submitted to the DataFax data management system at the IDI and was performed according to procedures in a detailed Data Management Operations Manual at the IDI. The study team and data entry staff reviewed source documents and laboratory reports to ensure accuracy and completeness. All forms were entered and verified according to standard operating procedures.

Statistical analyses were performed using STATA (Stata Corp. STATA 12.0, college station, Texas 77,845 USA). The investigational test was the modified Eiken TB-LAMP sputum test performed among presumptive TB participants, with results classified as positive or negative. A participant was considered TB-LAMP positive if any of sputum specimens tested positive. Continuous variables were summarized using medians and inter-quartile ranges (IQR) while categorical variables were summarized using frequencies, percentages and proportions. Wilcoxon rank sum test was used to compare continuous variables and either chi-square test or Fisher’s Exact test for categorical variables as appropriate. We compared the characteristics of the study population by the strata of MTB culture status.

To determine the performance of the modified Eiken TB-LAMP test in the diagnosis of TB among presumptive TB patients, a two by two table was used to calculate the sensitivity, specificity, positive and negative predictive values using MTB cultures as reference standard.

## Results

### Population characteristics

**Fig. 1:**
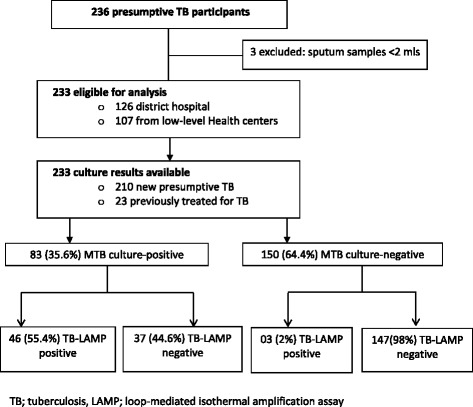
Participants flow chart

A total of 236 adult presumptive TB participants were enrolled, of which 233 were included in the analysis (Fig. 1). Three participants were excluded due to inadequate left-over sputum specimens. Of the 233 eligible participants; 44.6% (104/233) were females, median age 40 years (inter quartile range [IQR] of 30–53), 48.5% (113/233) were HIV-infected and 55.8% (63/113) were taking ART. A total of 210 (90.1%) participants were new presumptive TB while 23 (9.9%) had been previously treated for TB. The smear microscopy positivity rate was 18.5% (43/233) overall with 17.5% (22/126) among participants at Kiboga Hospital and 19.6% (21/107) among those at the low-level health centers. More than half (62.8%, 27/43) of the smear positives had bacillary load (acid fast bacilli) 2+ (25-250 AFB in one field) or 3+ (> 250 AFB in one field) (Table 1) with no significant difference in bacillary load between hospital and low-level health center settings (*p* = 0.867) (Table 2). Patients’ characteristics are shown in Table 1.

**Table 1:** Participant characteristics, comparing MTB-culture positives and negatives

Demographic and clinical characteristics	Total, *N* = 233 Frequency (%)	MTB Culture positive, *N* = 83	MTB Culture negative, *N* = 150	*P* value
Hospital setting	126 (54.1)	37 (44.5)	89 (59.3)	0.030
Median age, years (IQR)	40 (30–53)	38 (27-46)	41 (30-55)	0.240
Median time to LAMP testing, hours (IQR)	5 (4-6)	5 (5-6)	5 (4-6)	0.300
Gender				
Male	129 (55.4)	52 (62.7)	77 (51.3)	0.096
Female	104 (44.6)	31 (37.3)	73 (48.7)	
Cough for 2 weeks	232 (99.6)	83 (100)	149 (99.3)	1.000
Bloody sputum	34 (14.6)	12 (14.5)	22 (14.7)	0.966
Fever for 2 weeks	207 (88.8)	74 (89.2)	133 (88.7)	0.909
Night sweats for 2 weeks	180 (77.3)	67 (80.7)	113 (75.3)	0.347
TB treatment history				
Previous TB treatment	23 (9.9)	9 (10.8)	14 (9.3)	0.711
New TB treatment	210 (90.1)	74 (89.2)	136 (90.7)	
HIV co-infected	113 (48.5)	44 (53.0)	69 (46.0)	0.305
ART (*N* = 113)	63 (55.8)	23 (52.3)	40 (58.0)	0.552
Weight loss in 1 month	188 (81.0)	68 (81.9)	120 (80.5)	0.796
Smear microscopy				
Positive for AFB	43 (18.5)	38 (45.8)	5 (3.3)	0.000
*3+ (> 250 AFB in one field)*	*14*	*14*	*0*	
*2+ (25-250 AFB in one field)*	*13*	*12*	*01*	
*1+ (3-24 AFB in one field)*	*13*	*11*	*01*	
*Scanty (5-49 AFB in one length)*	*03*	*03*	*0*	
Negative for AFB	190 (81.6)	45 (54.2)	145 (96.7)	
TB-LAMP				
Positive	49 (21.0)	46 (55.4)	03 (2)	0.000
Negative	184 (79)	37 (44.6)	147 (98)	

*IQR* Interquartile range, *ART* Antiretroviral therapy, *AFB* Acid Fast Bacilli

**Table 2:** Sputum smear and LJ culture bacillary load by TB-LAMP test result and setting

	TB-LAMP		*P*-value	Site		*P*-value
	Positive, *N* = 49 (%)	Negative, *N* = 184 (%)		Hospital, *N* = 126 (%)	Health Centre, *N* = 107 (%)	
LJ MTB culture quantification (Colonies)						
Greater than 20 (*N* = 57)	43 (87.8)	14 (7.6)	0.000	26 (20.6)	31 (29)	0.061
10-20 (N = 2)	00	02 (1.1)		00	02 (1.87)	
Less than 10 (N = 1)	01 (2.0)	00		00	01 (0.9)	
Negative/contaminated (*N* = 173)	05 (10.2)	168 (91.3)		100 (79.4)	73 (68.2)	
Smear microscopy AFB quantification						
3+	14 (28.6)	00	0.000	07 (5.6)	07 (6.0)	0.867
2+	13 (26.5)	00		06 (4.8)	07 (6.5)	
1+	06 (12.2)	07 (3.8)		08 (6.4)	05 (4.7)	
Scanty	03 (6.1)	00		01 (0.8)	02 (1.9)	
Negative	13 (26.5)	177 (96.2)		104 (82.5)	86 (80.4)	

*AFB* Acid Fast Bacilli, *LJ* Lowenstein-Jensen

### MTB culture results

All 233 participants had valid MTB culture results and, thus, were eligible for the TB-LAMP performance analysis. Three participants that had contaminated LJ culture results had valid MGIT cultures while five participants that had contaminated MGIT culture results had valid LJ cultures. A detailed description of the TB culture results indicating bacillary load from LJ cultures is shown in Table 2. Of the 233 analyzable participants, 83/233 (35.6%) had one or more positive MTB cultures for *M. tuberculosis* complex by MGIT and/or LJ culture. A comparison of clinical characteristics between MTB culture-positive and negative participants is also shown in Table 1.

### Diagnostic performance of TB-LAMP assay compared to MTB culture as reference standard

TB-LAMP was positive among 49 (21%) of the 233 eligible participants. The median time of TB-LAMP testing was 5 h (IQR 4-6). Table 3 shows performance measures for sputum TB-LAMP test in the diagnosis of MTB using LJ and/or MGIT MTB cultures as reference standard. TB-LAMP was positive among 46 (55.4%) of the 83 MTB culture positive participants, majority (87.8%) of the TB-LAMP positives had high bacillary load (greater than 20 colonies) LJ culture (Table 2). Compared to MTB culture, overall sensitivity and specificity of TB-LAMP were 55.4% (95% CI 44.1-66.3) and 98.0% (95% CI 94.3-99.6) respectively in the diagnosis of PTB among presumptive TB adults. Among HIV-infected participants, TB-LAMP sensitivity and specificity were 52.3% (95% CI 36.7-67.5) and 97.1% (95% CI 89.9-99.6) respectively compared to MTB culture. TB-LAMP performance in the diagnosis of PTB determined using a combination of Xpert MTB/Rif test or MTB culture (any one of the tests positive) as reference standards is also shown in Table 3.

**Table 3:** Performance of TB-LAMP test in the diagnosis of TB determined using various reference standards

Population	Diagnostic performance measure	TB-LAMP performance % (95% CI)	
		LJ and /or MGIT MTB as reference standard	MTB culture and/or Xpert MTB/Rif test as reference
Overall performance, N = 233	Sensitivity	46/83, 55.4 (44.1-66.3)	47/95, 49.5 (39.1-59.9)
	Specificity	147/150, 98.0 (94.3-99.6)	136/138, 98.6 (94.9-99.8)
	PPV	46/49, 93.9 (83.1-98.7)	47/49, 95.9 (86.0-99.5)
	NPV	147/184, 79.9 (73.4-85.4)	136/184, 73.9 (66.9-80.1)
	LR (positive)	27.7 (8.9-86.4)	34.1 (8.5-137.2)
	LR (negative)	0.45 (0.36-0.58)	0.51 (0.42-0.63)
Smear-negatives, *N* = 190	Sensitivity	11/45, 24.4 (12.9-39.5)	11/56, 19.6 (10.2- 32.4)
	Specificity	143/145, 98.6 (95.1-99.8)	132/134, 98.5 (94.7-99.8)
	PPV	11/13, 84.6 (54.6-98.1)	11/13, 84.6 (54.6-98.1)
	NPV	143/177, 80.8 (74.2-86.3)	132/177, 74.6 (67.5-80.8)
	LR (positive)	17.72 (4.08 -77.0)	13.16 (3.01-57.47)
	LR (negative)	0.77 (0.65-0.91)	0.82 (0.72-0.93)
HIV-infected, N = 113	Sensitivity	23/44, 52.3 (36.7-67.5)	24/50, 48.0 (33.7-62.6)
	Specificity	67/69, 97.1 (89.9-99.6)	62/63, 98.4 (91.5-100)
	PPV	23/25, 92.0 (74.0-99.0)	24/25, 96.0 (79.6-99.9)
	NPV	67/88, 76.1 (65.9-84.6)	62/88, 70.5 (59.8-79.7)
	LR (positive)	18.0 (4.47-72.7)	30.24 (4.24-215.9)
	LR (negative)	0.49 (0.36-0.67)	0.53 (0.40-0.69)
Smear-negative HIV-infected, *N* = 96	Sensitivity	08/28, 28.6 (13.2-48.7)	08/33, 24.2 (11.1- 42.3)
	Specificity	67/68, 98.5 (92.1-100)	62/63, 98.4 (91.5-100)
	PPV	08/09, 88.9 (51.8- 99.7)	08/09, 88.9 (51.8- 99.7)
	NPV	67/87, 77.0 (66.8-85.4)	62/87, 71.3 (60.6-80.5)
	LR (positive)	19.43 (2.55-148.19)	15.27 (1.99-116.96)
	LR (negative)	0.72 (0.57- 0.92)	0.77 (0.63- 0.94)
Hospital, N = 126	Sensitivity	23/37, 62.2% (44.8-77.5)	23/42, 54.8 (38.7-70.2)
	Specificity	87/89, 97.8% (92.1-99.7)	82/84, 97.6 (91.7-99.7)
	PPV	23/25, 92.0 (74.0 -99.0)	23/25, 92.0 (74.0-99.0)
	NPV	87/101, 86.1 (77.8-92.2)	82/101, 81.2 (72.2-88.3)
Health centre III, N = 107	Sensitivity	23/46, 50.0% (34.9-65.1)	24/53, 45.3 (31.6-59.6)
	Specificity	60/61, 98.4% (91.2-100)	54/54, 100 (93.4-100.0)
	PPV	23/24, 95.8 (78.9-99.9)	24/24, 100.0 (85.8 -100.0)
	NPV	60/83, 72.3 (61.4-81.6)	54/83, 65.1 (53.8-75.2)

*PPV* Positive predictive value, *NPV* Negative predictive value, *LR* Likelihood ratio, *CI* Confidence intervals, *FM* fluorescence microscopy, *ZN* Ziehl-Neelson, *MGIT* mycobacterial growth indicator tube, *LJ* Lowenstein-Jensen

### TB-LAMP performance among smear-negative participants

Among the eligible participants, sputum smears were positive for AFB in 18.5% (43/233) participants; the remaining 190 participants were smear-negative and were used in the estimation of the performance of TB-LAMP among smear-negative participants. Among the 190 smear-negatives, 45 (23.7%) were MTB culture positive and were used to calculate sensitivity of TB-LAMP while 145 MTB culture-negatives were used to estimate specificity of TB-LAMP in this population of patients. Among the smear-negatives, TB-LAMP sensitivity and specificity were 24.4% (95% CI 12.9-39.5) and 98.6% (95% CI 95.1-99.8) respectively when compared to MTB culture as reference standard. Among the HIV-infected smear-negatives, the sensitivity and specificity were 28.6% (95% CI 13.2-48.7) and 98.5% (95% CI 92.1-100) respectively (Table 3). Performance of the TB-LAMP test among smear-negative patients was also determined using a combination of Xpert MTB/Rif test and MTB culture as reference standards are shown in the same Table.

### TB-LAMP performance in central hospital versus low-level health centre III’s

TB-LAMP sensitivity and specificity were 62.2% (95% CI 44.8-77.5) and 97.8% (95% CI 92.1-99.7) in the hospital setting where central testing occurred compared to 50.0% (95% CI 34.9-65.1) and 98.4% (95% CI 91.2-100) respectively in low-level health centers from which specimens were transported centrally (Table 3).

### TB-LAMP versus smear microscopy and Xpert MTB/Rif test performance

Performance indices of TB-LAMP, smear microscopy and Xpert MTB/Rif assay in the diagnosis of TB compared to MTB cultures as reference standard in this population of patients are shown in Table 4. TB-LAMP sensitivity was significantly higher than that of smear microscopy (ZN or FM methods) when MTB culture was used as reference standard, *p*-value =0.0325 (stratified McNemar’s test) with comparably high specificity, p-value =0.6875 (stratified McNemar’s test). TB-LAMP sensitivity was significantly lower than that of Xpert MTB/Rif test using MTB culture as reference standard, p-value =0.0215 (stratified McNemar’s test), however, the specificity was significantly higher than that of Xpert MTB/Rif test, p-value = 0.0225 (stratified McNemar’s test).

**Table 4:** Diagnostic performance of TB-LAMP, smear microscopy and Xpert MTB/Rif test in pulmonary TB

Diagnostic performance measure	Tests performance with LJ and /or MGIT MTB culture as reference standard		
	TB-LAMP performance (95% CI)	FM/ZN performance (95% CI)	Xpert MTB/Rif test performance (95% CI)
Sensitivity	46/83, 55.4 (44.1-66.3)	38/83, 45.8 (34.8-57.1)	54/83, 65.1 (53.8-75.2)
Specificity	147/150, 98.0 (94.3-99.6)	145/150, 96.7 (92.4-98.9)	138/150, 92.0 (86.4-95.8)
PPV	46/49, 93.9 (83.1-98.7)	38/43, 88.4 (74.9-96.1)	54/66, 81.8 (70.4-90.2)
NPV	147/184, 79.9 (73.4-85.4)	145/190, 76.3 (69.6- 82.2)	138/167, 82.6% (76.0-88.1)
LR (positive)	27.7 (8.9-86.4)	13.7 (5.62-33.6)	8.13 (4.62-14.31)
LR (negative)	0.45 (0.36-0.58)	0.60 (0.46-0.77)	0.38 (0.28-0.51)

*PPV* Positive predictive value, *NPV* Negative predictive value, *LR* Likelihood ratio, *CI* Confidence intervals, *FM* fluorescence microscopy, *ZN* Ziehl-Neelson, *MGIT* mycobacterial growth indicator tube, *LJ* Lowenstein-Jensen

### Operational suitability of TB-LAMP assay

The laboratory study personnel who performed the assay indicated that TB-LAMP test was easier to interpret than smear microscopy. The laboratory technician also noted that the complexity of TB-LAMP was comparable to that of smear microscopy. No significant errors were noted during the testing procedure and none of the tests required repetition. There were no major issues with the test other than long power outages which were managed by having a UPS connected to the TB-LAMP machine.

## Discussion

The modified Eiken TB-LAMP assay demonstrated higher sensitivity with similar specificity when compared to smear microscopy at point-of-need in a rural district in Uganda where smear microscopy is the available routine diagnostic option and power cutoffs are frequent. Our findings are in agreement with earlier reports on TB-LAMP evaluations which showed diagnostic accuracy higher than smear microscopy [11, 14, 15, 18, 21]. In the first multicenter clinical evaluation study on development and use of a TB-LAMP kit intended for peripheral laboratories by Boehme et al. 2007 performed in Peru, Bangladesh and Tanzania [16], the overall TB-LAMP sensitivity in detection of MTB was 88.2% (95% CI 83.9-92.5%) while the specificity was 99.0% (95% 98.1-99.9%) [16]. Boehme’s study also reported TB-LAMP sensitivity among smear-negative, culture-positives of 48.8% (95% CI 33.9-63.7%). Following earlier reports on the advantages of TB-LAMP over the existing TB tests as a potential point-of-need test, several modifications including better sensitive primers and less technical complexity have been made to the earlier versions of the TB-LAMP most of which have shown promising accuracy results [11–15, 18]. When compared to the performance of TB-LAMP reported by WHO [10], our study demonstrated a significantly lower sensitivity; however, the specificity was high and consistent with previous studies [12, 15, 16, 18].

The lower sensitivity demonstrated in our study could be due to the high prevalence of HIV in our study population; almost 50% of the studied population was HIV co-infected. Similar to earlier reports [10, 15] the overall sensitivity of TB-LAMP among HIV co-infected patients was low, probably due to the paucibacillary nature of TB in HIV co-infection as well as a higher prevalence of extra-pulmonary TB. Lack of CD4 cell count information in the study limited our ability to assess the sensitivity of TB-LAMP by CD4 count in HIV-infected participants. The other possible explanation for the low sensitivity of TB-LAMP assay shown in our study is specimen type and handling. Our study used left-over sputum from routinely collected sputum specimens which could have diminished the quality of the sputum tested and thus the low sensitivity. In addition, specimens from the low-level health centers were transported to a hub in the district hospital and had a lower sensitivity compared to specimens collected at the central hospital. The transport delay could have affected the quality of sputum used for TB-LAMP testing. Ultimately, only a single technician performed the tests during the entire study period and the technician was responsible for setting up the test room which could have caused delays in procedure performance and could have affected the sensitivity of the test. There is earlier evidence that the TB-LAMP test is operator-dependent [16]. Furthermore, several other studies performed in peripheral settings had also demonstrated lower sensitivity of TB-LAMP compared to WHO reported values [10, 21]. Test environment including dust and humidity could have adversely affected the sensitivity of TB-LAMP test in this rural setting. Lastly, our findings showed that majority of the TB-LAMP positives also had high bacillary load on sputum smear (AFB 2+ and 3+) and LJ culture (greater than 20 colonies). It is therefore possible that TB-LAMP will only detect TB in sputum specimens with high bacillary load but not low bacillary load specimens, thus the low sensitivity found in our study.

Although TB-LAMP sensitivity was better than that of smear microscopy, when compared to Xpert MTB/Rif test, TB-LAMP showed lower sensitivity but higher specificity. The findings are in agreement with earlier reports that compared TB-LAMP with Xpert MTB/Rif test [14, 22]. The possibility of Xpert MTB/Rif test to detect dead bacilli and DNA in patients may account for the lower specificity observed when compared to TB-LAMP test.

A strength to our study is the comparison of the TB-LAMP test with the routine tests such as smear microscopy and Xpert MTB/Rif test, however, we acknowledge the limitation that we did not compare the TB-LAMP to urine TB Lipoarabinomannan (LAM) test as at the time of this diagnostic evaluation study, urine TB LAM test was only available in research settings. Another strength to our study is the performance of both MGIT and LJ TB cultures on the samples, which strengthened the reference standard to which TB-LAMP was compared in this study. However, use of left-over expectorated sputum specimens could have reduced the quality of sputum tested thereby affecting the results.

Overall, from our evaluation, TB-LAMP procedure was found easier and faster compared to the conventional available TB tests. There was no need for sophisticated infrastructure or intensive training. These findings were similar to earlier reports on the TB-LAMP test.

## Conclusions

In this high HIV/TB prevalence rural setting, TB-LAMP performs better than conventional smear microscopy in the diagnosis of MTB among presumptive TB patients although the sensitivity is lower than that reported by the World Health Organization. TB-LAMP can easily be performed following a short training period and in absence of sophisticated infrastructure and expertise. TB-LAMP maybe useful in centers where Xpert MTB/Rif test is not available and microscopy is the only option. Given its low, TB-LAMP may have limited use in the diagnosis of TB among HIV co-infected individuals.
